# Supplementary material for: Arabidopsis Trichome Contains Two Plasma Membrane Domains with Different Lipid Compositions Which Attract Distinct EXO70 Subunits
Source: Int J Mol Sci. 2019 Aug 3;20(15):3803. doi: 10.3390/ijms20153803 (PMC6695903; doi:10.3390/ijms20153803)
Supplement: Supplementary file 1 [file ijms-20-03803-s001.zip › ijms-543215-supplmentary.pdf]

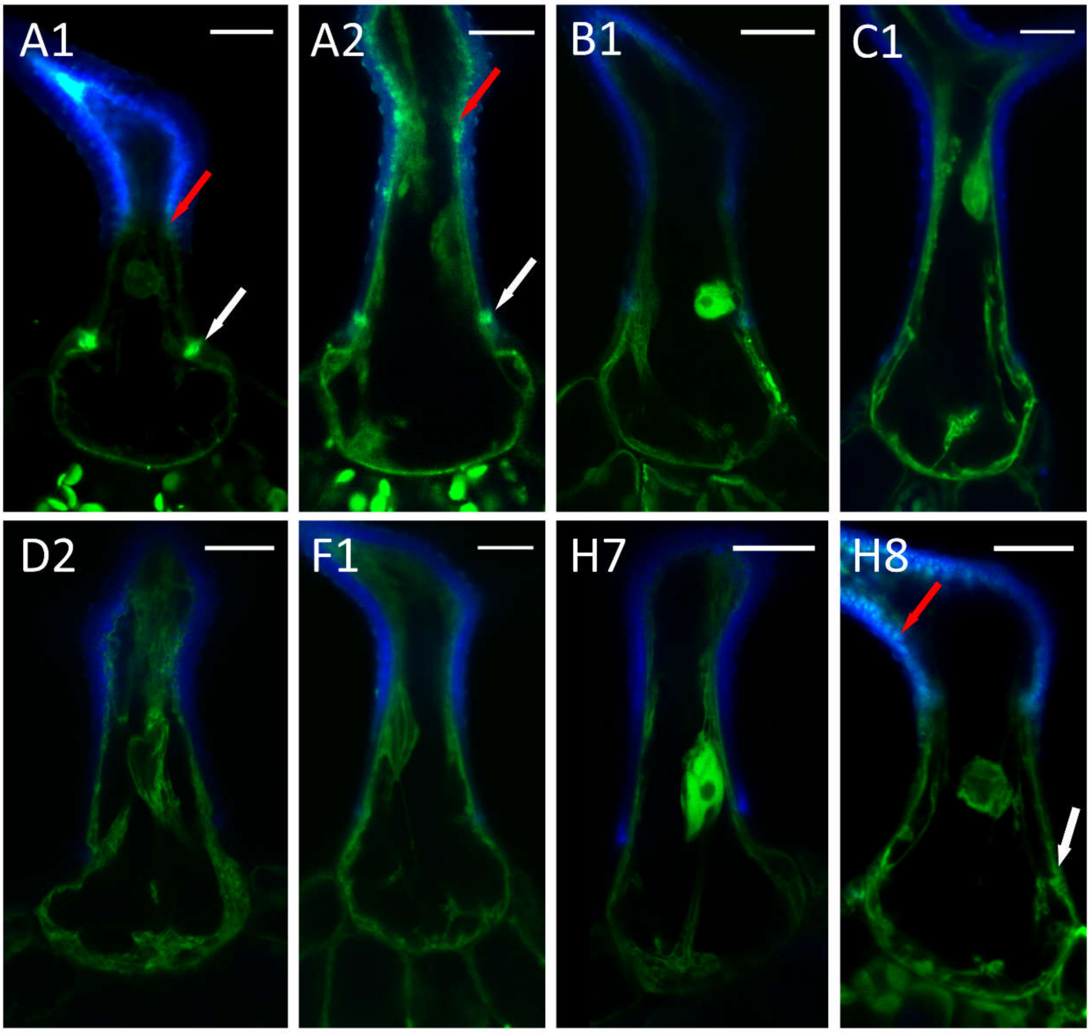

Supplemental Figure S1. Localization of different EXO70s in WT trichome: blue—cell wall autofluorescence, green—GFP, white arrows point at the Ortmannian ring, red arrows point at examples of apical domain accumulations; Scale bars = 20 μm.

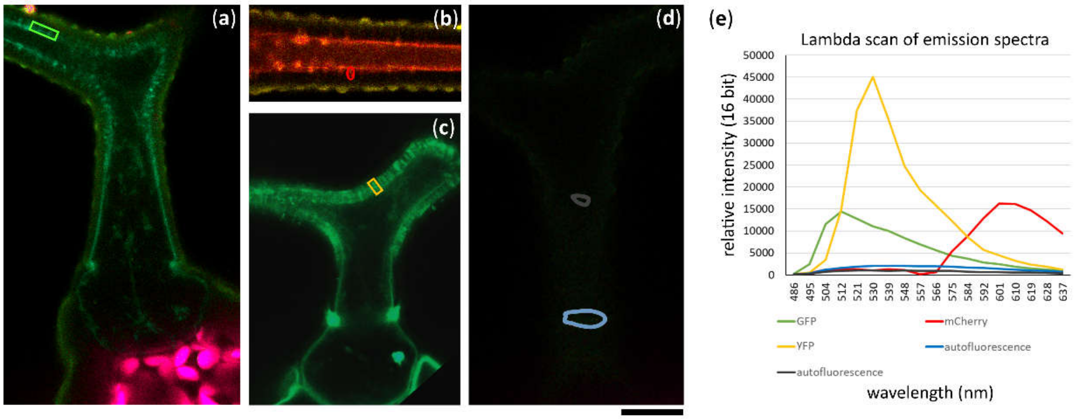

Supplemental Figure S2. Lambda scan controls of fluorescent markers entrapped in the SCW of WT trichome: (**a**) GFP-EXO70H4; (**b**) mCh-EXO70H4; (**c**) YFP-PA marker; (**d**) negative control, untransformed WT plant; (**e**) lambda scan of emission spectra; squares depict measured area; images are a projection of wavelength series, where each channel was given corresponding color; Scale bar = 20 μm.

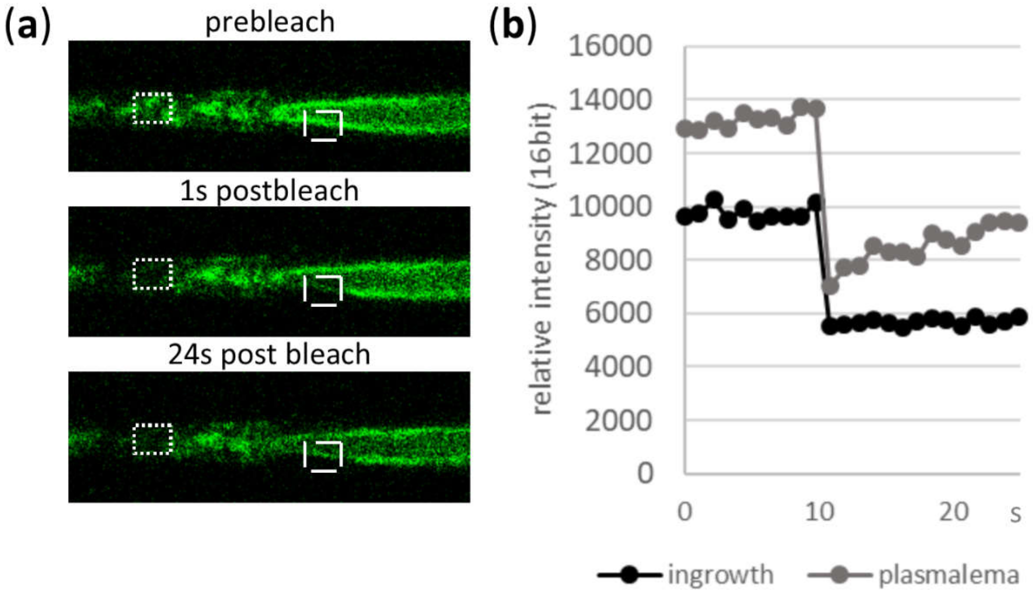

Supplemental Figure S3. FRAP of the PI4P marker in the active PM and intramural pockets: (**a**) PI4P marker in the mature trichome branch. Dotted rectangle represents the region with entrapped membranous pockets, dashed rectangle represents the active cytoplasm region. ; (**b**) Relative intensity of both regions (ingrowth corresponds to the dotted rectangle, PM to the dashed rectangle). Bleaching was performed 9 s after the beginning of the imaging.

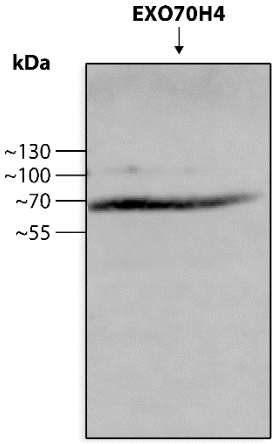

Supplemental Figure S4. Western blot analysis of N-terminally HA-tagged EXO70H4 protein expression and stability.

Supplemental Table S1. List of used primers.

EXO70A1 prom for	TTGTACAAACTTGAAAAATAACGAATAATCTTTCTGAGTTGAG
EXO70A1 prom rev	ATAGAAAAGTTGAAAATTACATTACTGGTTGATGGAAAG
PKI_NES-GA5-F_Xba	ATATCTAGAAACTCAAATGAACTTGCCCTAAAACTTGCAGGGCTCGATATTA ACAAGACCGAGGGTGCTGGTGCTGGTGCTGGTGCTGGTGCCGGCATG
Spo20-R_SpeI-int	ACCACTAGTCTTAGTGGCGTCATCGAAC
EXO70H4-forward	TTCCAGATTACGCTATGATGACGAGAAAAGC
EXO70H4-reverse	TAGCGGCCGCTTAGGACATGGATTGC
megaprimer with HA-tag and Kozak seq.	AAGTCGACGCCGCCACCATGTACCCATAC GATGTTCCAGATTACGCTATG
